# Effects of Simulated Interventions to Improve School Entry Academic Skills on Socioeconomic Inequalities in Educational Achievement

**DOI:** 10.1111/cdev.12309

**Published:** 2014-10-18

**Authors:** Catherine R Chittleborough, Murthy N Mittinty, Debbie A Lawlor, John W Lynch

**Affiliations:** 1 University of Adelaide; 2 University of Bristol

## Abstract

Randomized controlled trial evidence shows that interventions before age 5 can improve skills necessary for educational success; the effect of these interventions on socioeconomic inequalities is unknown. Using trial effect estimates, and marginal structural models with data from the Avon Longitudinal Study of Parents and Children (*n *=* *11,764, imputed), simulated effects of plausible interventions to improve school entry academic skills on socioeconomic inequality in educational achievement at age 16 were examined. Progressive universal interventions (i.e., more intense intervention for those with greater need) to improve school entry academic skills could raise population levels of educational achievement by 5% and reduce absolute socioeconomic inequality in poor educational achievement by 15%.

Socioeconomic disadvantage in childhood is associated with reduced ability to benefit from schooling (Hertzman & Power, 2003; Lynch, Law, Brinkman, Chittleborough, & Sawyer, 2010), poorer educational outcomes throughout schooling (Dearing, McCartney, & Taylor, 2009; Goodman, Gisselmann, & Koupil, 2010), a lower likelihood of continuing to tertiary education (Bynner, Joshi, & Tsatsas, 2000; Goodman et al., 2010), and less labor market success (Fasih, 2008). Poorer educational outcomes have been associated with increased welfare dependence (Pape, Bjorngaard, Westin, Holmen, & Krokstad, 2011) and lower skilled jobs with lower median hourly pay rates (Office for National Statistics, 2011).

This article uses observational data from a large British birth cohort study, combined with effect estimates observed in randomized and quasi-experimental trials, to address the question of what would happen to population levels of, and socioeconomic inequalities in, educational achievement if school readiness could be improved through effective interventions. School readiness can include a broad range of health and development characteristics, including physical health, social and emotional well-being, and academic and personality skills such as attention and self-regulation abilities (Heckman, Pinto, & Savelyev, 2013). In this study, we use teacher-rated academic skills as the measure of school readiness because these are relatively direct precursors of our academic achievement outcome measured at ages 15–16. Early academic skills have been shown to be stronger predictors of later school reading and math achievement than attention and socioemotional abilities (Duncan et al., 2007).

Inequalities in cognitive, behavioral, and social assets gained in early childhood influence socioeconomic pathways into adulthood and inequalities in health later in life (Lynch & Smith, 2005). Differences in cognitive outcomes across socioeconomic groups have been observed from the first 2 years of life (Fernald, Kariger, Hidrobo, & Gertler, 2012; Schoon, Hope, Ross, & Duckworth, 2010), with the gap between the highest and lowest socioeconomic groups widening through the school years (Goodman, Gregg, & Washbrook, 2011). Early economic hardship and social adversity undermine many aspects of child health and development, including physical and mental health, cognitive development, and educational achievement (Kaplan, Turrell, Lynch, Everson, & Helkala, 2001; Shonkoff, Richter, van der Gaag, & Bhutta, 2012), which are strongly associated with health and well-being in adulthood (Galobardes, Lynch, & Smith, 2008; Lynch, Kaplan, & Salonen, 1997). Policies and interventions that improve capabilities for productivity and participation are essential for improving health and reducing inequalities (Di Cesare et al., 2013). “Giving every child the best start in life” and “enabling all children, young people and adults to maximize their capabilities” have therefore been identified as key actions to reduce social inequalities in health (The Marmot Review, 2010).

## Effectiveness of Early Childhood Interventions

There is strong evidence that early investments in child care and preschool to improve abilities and skills prior to formal schooling provide long-term benefits in promoting future academic achievement and productivity in adulthood (Heckman, 2008; Shonkoff, 2012). It is recommended that such interventions be provided under a policy of “progressive universalism” with universal services provided for all families, and progressively more intensive support targeted toward families with greater need (Lynch et al., 2010; The Marmot Review, 2010). There is little empirical and conceptual research on this intuitively appealing idea, but obtaining the right mix of universal and targeted services, and who is eligible for intensive support, is key to any potential scaling up of evidence-based early childhood programs (Duncan & Sojourner, 2013; Lynch et al., 2010).

The largest effects on development have been demonstrated in randomized trials of the Abecedarian and High Scope/Perry Preschool programs (Duncan & Magnuson, 2013), which were high-quality child-care and preschool interventions for low-income African American children (Currie, 2001). Other randomized studies demonstrating positive effects on cognitive and academic ability include the Chicago School Readiness Project (Raver et al., 2011) and a nurse home visiting program (Olds et al., 2004). Studies have also examined effects of more typical preschool programs, although this evidence is limited by the lack of randomized experiments. Further description of these studies is included in Appendix S1 (all appendices referenced in this article are in the online Supporting Information).

## Effect of Interventions on Average Levels and Inequalities in Educational Achievement

While the studies mentioned earlier provide evidence that early childhood interventions can improve school readiness and academic outcomes, they do not provide information about how such interventions may affect population levels of, and socioeconomic inequalities in, educational outcomes. In other words, they do not provide information on what happens when such interventions are “scaled up” within a progressive universal framework for service provision. The only work of this type of which we are aware is an innovative recent analysis by Duncan and Sojourner (2013) who used effect estimates from the Infant Health and Development Program (IHDP; Gross, Spiker, & Haynes, 1997) to simulate what would happen to income-based achievement gaps if a similar Abecedarian-type program to IHDP were applied to the U.S. population. Their results suggest that income-based achievement gaps in IQ would essentially be eliminated under either a universal or targeted IHDP-type intervention at age 3 and substantially reduced at ages 5 and 8.

The key question in this study is to estimate the effects of improving school entry academic skills at age 5 on educational achievement at ages 15–16, and how this would affect socioeconomic inequality in educational achievement. In other words, how much can socioeconomic inequality (exposure, *X*) in educational achievement (outcome, *Y*) be reduced by improving school entry academic skills (mediator, *M*), and what effect would this have on overall population levels of educational achievement? Gaining insights into such questions may help illustrate the limits of what social policy around early child development interventions may be able to achieve at the population level. The causal diagram for these associations is depicted in Appendix S2. We used a marginal structural model to overcome limitations of other techniques as discussed in Appendix S2.

For our first study objective, we estimated the controlled direct effect of socioeconomic disadvantage on adolescent educational achievement that is not mediated by school entry academic. The second objective of the study simulates the effects of plausible evidence-based universal and targeted interventions to improve school entry academic skills on poor educational achievement and socioeconomic inequality in adolescent educational achievement. We use data from a richly characterized, large birth cohort in the United Kingdom—the Avon Longitudinal Study of Parents and Children (ALSPAC)—and effect estimates of interventions tested in randomized controlled trials.

## Method

### Sample

ALSPAC is a prospective, geographically representative study of children born to women resident in the Avon area of southwest England with an expected delivery date between April 1, 1991, and December 31, 1992. The core ALSPAC sample consists of 14,541 pregnancies that resulted in 14,676 known fetuses, of which 14,062 were live births and 13,978 children alive at 1 year (Boyd et al., 2013; Fraser et al., 2013). The 14,541 pregnancies represent 72% of the eligible pregnancies in the region during this period (Boyd et al., 2013). The ALSPAC data are valuable because of the large number of covariates that can be used to control for confounding, the length of follow-up that includes recent education data at ages 15–16, and the broadly representative nature of the sample. The 1991 census was used to compare mothers with infants < 1 year in the ALSPAC sample to those living in the Avon region and those in the whole of Britain (Fraser et al., 2013), and few differences were found. Mothers in ALSPAC at 8 months postpartum were more likely to live in owner-occupied housing (79% ALSPAC, 69% Avon, 63% Britain), to have a car in the household (91% ALSPAC, 84% Avon, 76% Britain), to be White (98% ALSPAC, 96% Avon, 92% Britain), and to be married (79% ALSPAC, 72% Avon, 72% Britain), but less likely to live in overcrowded conditions (34% ALSPAC, 26% Avon, 31% Britain; Fraser et al., 2013). For confidentiality reasons, data on the 13 triplet and quadruplet children were not available for analysis. The study website contains details of all the data that are available through a fully searchable data dictionary (http://www.bris.ac.uk/alspac/researchers/data-access/data-dictionary/). Ethical approval was obtained from the ALSPAC Ethics and Law Committee and the Local Research Ethics Committees.

### Educational Achievement

In the United Kingdom, the General Certificate of Secondary Education (GCSE) is the main qualification taken by 14- to 16-year-olds when they complete the first stage of secondary school education (Key Stage 4). Sixteen years is the minimum age at which children can legally end their education, and education beyond this age is referred to as higher or further education. For this study, poor educational achievement was defined as not achieving five or more A* to C grade GCSEs or equivalent, including English and Maths, which has been set as a national improvement target by the U.K. government (Department for Communities and Local Government, 2008). This level of school achievement is a key prerequisite for further study in advanced (A) levels and at higher education institutions, and for gaining access to prestigious apprenticeships and skilled employment (Unwin, 2010; Wolf, 2011). The mean age of ALSPAC children at the start of the academic year in which most will have sat their final GCSE examinations was 15.5 years (interquartile range = 15.0–16.8). Data were obtained through linkage to the National Pupil Database (NPD) for England, using name, date of birth, and postcode of the cohort members matched to Unique Pupil Number. Key Stage 4 records were identified for 84% of children alive at 1 year in the core ALSPAC sample. It is not possible to link children not attending government- maintained schools, for example, those attending private schools or being home educated, to the NPD. Children may also not be linked because of leaving the United Kingdom, changing their family name, or errors in personal details within the NPD. Children in ALSPAC have been shown to have a higher Key Stage 4 academic achievement score (*M* = 317) than pupils in all English government schools (*M* = 308; Boyd et al., 2013). Pupils enrolled in ALSPAC were also more likely to be White (96%) and less likely to be eligible for free school meals (6%) than pupils in all English government schools (87% and 13%, respectively; Boyd et al., 2013).

### Early Life Socioeconomic Disadvantage

To capture the multidimensionality of socioeconomic position (Galobardes, Lynch, & Smith, 2007; Lynch & Kaplan, 2000) a Socioeconomic Index was computed using latent class analysis with PROC LCA in SAS (Lanza, Collins, Lemmon, & Schafer, 2007). The latent class model identified underlying subgroups of individuals who share a variety of socioeconomic characteristics. Six socioeconomic variables recorded in the parent - or caregiver-completed questionnaires during pregnancy were used to calculate the Socioeconomic Index: The highest of mother's or partner's highest education level (degree or higher; A level, the advanced level examinations taken 2 years after O level and usually required for university entry; O level, the ordinary level examinations most commonly taken at 16 years; less than O level), the highest of mother's or partner's social class (I or II, professional, managerial and technical; III, skilled manual or nonmanual; IV or V, semiskilled or unskilled manual) based on the Registrar General's classification of occupations (Office for National Statistics, 1991), home ownership (owned or mortgaged; rented or other), household crowding (0.5 or less; > 0.5 to 0.75; > 0.75 to 1; > 1 person per room), mother or partner unemployed or seeking a job (yes; no), and financial difficulties. Financial difficulties were assessed from five questions asking how difficult the mother found it to afford food, clothing, heating, rent or mortgage, and things she will need for the baby, with a score of 1 (*very difficult*) to 4 (*not difficult*) for each response. The algorithm for calculating the overall score was 20 minus the sum of the scores of each of the five items, where 0 represented no financial difficulties and 15 the maximum financial difficulties. Participants scoring > 8 were defined as experiencing financial difficulties (Bowen, Heron, Waylen, & Wolke, 2005). All six indicators of socioeconomic position had similar individual effects on school entry academic skills and poor educational achievement (see Appendix S3).

Three classes of socioeconomic position were identified from the LCA, which computes the posterior probability of an individual's membership in each class. Each individual was assigned to the class for which they had the highest probability. Two-, three-, and four-class models were tested, but Akaike's information criterion indicated that the three-class model was the best fit. Face validity of these three classes was demonstrated through analyses that showed participants in the “high” socioeconomic class were more likely to have a degree-level education, be in Social Class I or II, have 0.5 people or less per room, own their dwelling or have a mortgage, and less likely to experience financial difficulties or unemployment. Participants in the “medium” socioeconomic class were more likely to have A level or O level education, be in Social Class III, have more than 0.5 people but < 0.75 people per room, own their dwelling or have a mortgage, and less likely to experience unemployment. Participants in the “low” socioeconomic class were more likely to have less than O level education, be in Social Class IV or V, have more than 0.75 people per room, be renting their dwelling, and experience financial difficulties or unemployment.

### School Entry Academic Skills

School entry academic skills of children were rated by their teacher in the first half of their first term in reception class, aged 4–5 (South Gloucestershire Professional and Curriculum Support Service, 1996). While the Early Years Foundation Stage Profile and its predecessors were not in place at this time, a working group of heads, teachers, early years advisers, and educational psychologists within the Local Education Authorities in the Avon area had developed a baseline observational assessment undertaken by reception class teachers (Meadows, Herrick, & Feiler, 2007; Roulstone, Law, Rush, Clegg, & Peters, 2011). Teachers scored children on reading, writing, language, and maths. Scores for ALSPAC participants were obtained through parent-consented record linkage. School entry assessment data were linked for 68% of children alive at 1 year. Records were unable to be linked for 32% of the sample because local education authorities were only able to provide data for approximately 80% of schools as some schools did not complete the assessments. In addition, some children had moved out of the Avon area before starting school, and a minority had family name changes or incorrect personal details that prevented linkage. Integer scores between 2 and 7 on each of four required scales (language, reading, writing, maths) were summed to give a total score. The total school entry assessment score was divided into quintiles. Children scoring in the top two quintiles (scores 15–20) were defined as having a high score, those scoring in the third and fourth quintiles (scores 11–14) as medium, and those in the lowest quintile (scores 10 or lower) as low. Early math ability has been shown to have stronger associations with later reading and math achievement than early reading ability (Duncan et al., 2007), but as our later educational achievement outcome includes both English and maths, we have not separated math and literacy at school entry.

### Confounders of Association Between Socioeconomic Disadvantage and Educational Achievement

Confounders of the effect of early life socioeconomic disadvantage (*X*) on the outcome (*Y*) included parental ethnicity, assessed by self-completed questionnaire at 32 weeks gestation and coded as non-White if either parent identified as being non-White, and maternal age at last menstrual period prior to recognition that she was pregnant.

### Confounders of Association Between School Entry Academic Skills and Educational Achievement

Child sex was included in models as a confounder of the effect of school entry assessment (*M*) on the outcome (*Y*), not caused by socioeconomic disadvantage (*X*). The age difference between when school entry assessment and GCSE were assessed was 130 or 131 months for 99.3% of children with data for both measures (*n *=* *8,629). This indicates that if a child was relatively young at school entry assessment, they were also relatively young when they did their GCSE. Age at school entry assessment was therefore included as a confounder of the effect of *M* on *Y*, dichotomized at the median into < 55 months and 55 months or older.

The following variables were considered confounders of the effect of school entry assessment (*M*) on the outcome (*Y*), caused by early life socioeconomic disadvantage (*X*). Gestational age was calculated based on mother's last menstrual period. If last menstrual period was unknown, or considered unreliable, dating was based on clinical records of the earliest ultrasound scan or pediatric or obstetric assessment of the newborn. Preterm infants were those born before 37 weeks gestation. Birth weight was obtained from obstetric records and standardized (*z* score) for gestational age and sex using a standard reference population (Cole, Williams, Wright, & RCPCH Growth Chart Expert Group, 2011). Duration of breastfeeding, including both exclusive and nonexclusive, was assessed in the questionnaire when the child was 6 months old and coded as *never*, < *1* *month*, *1 to* < *3* *months*, 3 *to* < 6 *months*, or *at least 6 months*. Poor attachment was assessed by the question, “Very occasionally, mothers have mentioned that they felt quite unattached to their babies or even that they felt dislike for them for several weeks. Has this ever happened to you?” included in the questionnaire when the child was 47 months old. If mothers responded positively, they were classified as having feelings of poor attachment. Average weekly take-home family income (> £400, £300–£399, £200–£299, £100–£199, < £100) and marital status (first marriage, second or third marriage; separated, divorced, widowed; never married) were also assessed in this questionnaire. Maternal depression was assessed using the Edinburgh Postnatal Depression Scale when babies were 8 weeks old (Cox, Holden, & Sagovsky, 1987). Each question had four response categories scored from 0 to 3 and referred to the feelings of the mother in the past week. A score above 12 indicated probable depressive disorder (Cox et al., 1987). Whether mothers had smoked regularly since birth (yes; no) was also assessed in this questionnaire. An adaptation of the Home Observation for the Measurement of the Environment (Caldwell & Bradley, 1979) included in the questionnaire at 18 months used six questions related to whether the child had cuddly, push or pull, or coordination toys; the number of books they owned; and the frequency the mother tried to teach the child or talk to the child while she was doing housework or occupied in some other way. Summing the responses to these questions resulted in a score ranging from 0 to 12.

When children were 18 months old, parents reported on how they would assess the health of their child over the past year. The four-item responses were dichotomized into very healthy, no problems, or healthy but a few minor problems versus sometimes quite ill or almost always unwell. Child developmental abilities at 18 months were assessed using the ALSPAC developmental scale, created using items derived from the Denver Developmental Screening Test shown to be most predictive of developmental abnormality (Frankenburg, Dodds, Archer, Shapiro, & Bresnick, 1992). Many Denver items were designed to be observed by trained examiners so it was adapted for parental report after focus group piloting with the ALSPAC cohort. Parents reported whether their child could do 56 activities within four developmental domains (gross motor, fine motor, communication, and social skills). The number of passes, indicated by, “yes can do well,” was summed in each of the four subscales, and the total development score was summed across subscales. Age for completion was restricted to an 8-week window around 18 months given the developmental age-specific nature of the questions (Heron, Golding, & ALSPAC Study Team, 2004).

A total behavioral difficulties score was created by summing the scores of the hyperactivity, emotional symptoms, conduct problems, and peer problems scales of the Strengths and Difficulties Questionnaire (SDQ) parent version (Goodman, 1997), which was completed by the main caregiver, usually mother, when their child was 47 months old, using a scale from 1 to 3 (*does not apply, applies somewhat, definitely applies*).

Ten questions assessing parental warmth and hostility, including how often the mother shouts at or smacks the child during temper tantrums, and how often the child is praised or cuddled, were assessed in questionnaires when the child was aged 18–42 months. Fourteen questions assessing parental control, including the degree of choice the child is given with meals and clothing, how often they are allowed to stay up after bedtime or have dessert when they refused their main meal, and how often the parent has battles of wills with the child or reasons with them during tantrums, were included in questionnaires when the child was aged between 18 and 47 months. LCA identified three parental hostility groups based on their frequency of harsh discipline, and three parental control groups defined as high reasoning (highest frequency of reasoning and most choice), medium reasoning (less frequent reasoning and limited choice), and low reasoning (least frequent reasoning and limited choice).

### Analysis

Descriptive statistics were computed for all variables by socioeconomic disadvantage. To examine the effect of socioeconomic disadvantage and school entry academic skills on poor educational achievement, we used a standard regression of poor educational achievement (*Y*) separately on early life socioeconomic disadvantage (*X*) and school entry assessment (*M*) adjusted for measured confounders (*C*) to provide the total effects of both the exposure and mediator (Model 1). In Model 2, we assessed the controlled direct effect of socioeconomic disadvantage on educational achievement with a marginal structural model of the form

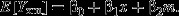


Assuming that there is no unmeasured confounding (VanderWeele, 2009), the controlled direct effect (relative risk [RR]) comparing *X*_*i*_ = *x* with *X*_*i*_ = x* is

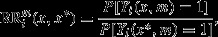
 where *Y*_*i*_(*x,m*) represents the counterfactual outcome *Y*_*i*_ if, counter to fact, *X*_*i*_ had been set to *x* and *M*_*i*_ had been set to *m*. No interactions were observed between school entry academic skills and socioeconomic disadvantage (*p *>* *.8) or gender (*p *>* *.3) using likelihood ratio tests so interaction terms were not included in models. We fitted a weighted generalized linear regression model

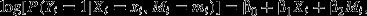
 with stabilized inverse probability weights of the form 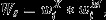 where

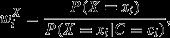
 and

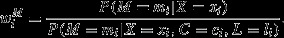


As described previously (Nandi, Glymour, Kawachi, & VanderWeele, 2012), the coefficient β_1_ is the controlled direct effect of early life socioeconomic disadvantage on educational achievement that does not act through school entry academic skills but may act through other factors, *L*, such as child characteristics, parenting, and home environment. Weights were estimated using multinomial logistic regressions for socioeconomic disadvantage and school entry assessment. For comparison purposes we also conducted a conventional regression model adjusting early life socioeconomic disadvantage (*X*) for school entry assessment (*M*), and measured confounders *C* and *L* (Model 3).

Using a marginal structural model in this observational study to determine the causal effect of socioeconomic disadvantage on poor educational achievement assumes no unmeasured confounding. We conducted sensitivity analyses (described in Appendix S4) to assess the effect of an unmeasured confounder of *X* and *Y*, on the controlled direct effect of socioeconomic disadvantage on poor educational achievement.

We also used marginal structural models to examine the effect of four hypothetical interventions to improve school entry assessment. The four hypothetical interventions reflect plausible effects obtained from randomized controlled trials and quasi-experimental trials. Intervention Scenario 1 was a universal program that improved the school entry assessment score equally for all children by 0.2 *SD*. This effect size is typical of those reported in previous studies, for example, the Effective Provision of Pre-school Education (Sammons et al., 2004), the Early Childhood Longitudinal Study's Kindergarten Class (ECLS–K) study (Magnuson, Ruhm, & Waldfogel, 2007), the National Institute of Child Health and Human Development (NICHD) longitudinal study (NICHD Early Child Care Research Network, 2002), and prekindergarten in five U.S. states (Wong, Cook, Barnett, & Jung, 2008). Intervention Scenario 2 was consistent with a progressive universal approach to intervention with a proequity effect favoring socioeconomically disadvantaged children. This means that all children receive a preschool program and disadvantaged children are provided with extra support and a more intensive program. Intervention Scenario 2 improved the school entry score of children with low socioeconomic position by 0.8 *SD* and other children by 0.2 *SD*. These effect sizes are consistent with the Oklahoma Pre-Kindergarten Program (Gormley, Gayer, Phillips, & Dawson, 2005; Karoly, Kilburn, & Cannon, 2005) and the ECLS–K study (Magnuson et al., 2007). Intervention Scenario 3 was a perverse universal program with an effect favoring children of high socioeconomic position, as when children with high socioeconomic position have better access to and better resources to exploit high-quality programs (Magnuson & Shager, 2010; Victora, Vaughan, Barros, Silva, & Tomasi, 2000). This involved improving the school entry score of children with high socioeconomic position by 0.8 *SD* and others by 0.2 *SD*. Intervention Scenario 4 was a proequity targeted program improving school entry score by 0.8 *SD* only among children with low socioeconomic position, as seen with the Abecedarian and High Scope/Perry Preschool programs (Campbell, Pungello, Miller-Johnson, Burchinal, & Ramey, 2001; Karoly et al., 2005) with no effect on the rest of the population. Further detail of the studies informing these scenarios and the effect sizes is described in Appendix S1. The marginal structural models take the form

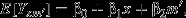
 and predict the expected outcome had there been a hypothetical intervention, such as Scenarios 1–4, to change *M* to a specific value *m′*.

### Multiple Imputation

Missing data due to attrition and noncompletion of questions may introduce bias in longitudinal studies if associations under investigation differ between respondents and nonrespondents. Of the 11,764 respondents with data on the GCSE outcome, 3.3% had missing data on socioeconomic position and 26.8% had missing data on school entry assessment. Multiple imputation was used to account for the potential bias from missing data. Multiple imputation by chained equation was used to impute missing data using the “mi impute chained” command in Stata version 12.0 (StataCorp, College Station, TX). Missing data were imputed on the outcome, exposure, mediator, and confounding variables for respondents who were alive at 1 year (*n *=* *13,978), and results reported for those with complete outcome data (*n *=* *11,764). Details of the imputation are described in Appendix S5. Analyses conducted using participants with complete data on all outcome, exposure, mediator, and confounder variables (*n *=* *4,290) are presented in Appendix S6. A flow chart of the analysis samples is provided in Appendix S7.

## Results

A description of all study variables in the response sample, complete cases, and imputed sample is listed in Table1. Tables1 and 2 show that overall 49.5% CI [48.6, 50.4]) of children had poor educational achievement at ages 15–16, although this ranged from 22.4% among socioeconomically advantaged children to 73.6% among socioeconomically disadvantaged children. The proportion with a low school entry assessment score was 23.9% (95% CI [23.0, 24.7]) and ranged from 9.8% among socioeconomically advantaged children to 39.0% among socioeconomically disadvantaged children. Among children with higher school entry scores, 24.2% had poor educational achievement, compared to 78.2% among those with low school entry scores.

**Table 1:** Distributions of Outcome, Exposure, Mediator, and Confounder Variables in Those With Observed Data, Complete Cases, and the Imputed Sample

	Response sample		Complete cases *n* = 4,290	Imputed sample *n *= 11,764
	*n*	% or (*M*)	% or (*M*)	% or (*M*)
Outcome				
Poor educational achievement	11,764	49.5	41.9	49.5
Exposure				
Socioeconomic Index	13,482			
High		25.1	24.1	21.9
Medium		45.9	55.5	48.6
Low		29.0	20.4	29.5
Mediator				
School entry assessment	9,461			
High		30.6	37.2	31.9
Medium		44.3	44.2	44.3
Low		25.1	18.6	23.9
Confounders of *M*-*Y* caused by *X*				
Birth weight, g	13,867	(3,394.3)	(3,433.2)	(3,388.9)
Preterm birth, < 37 weeks	13,978	5.7	4.6	5.8
Breastfeeding, never	11,141	24.7	24.2	28.2
Breastfeeding, at least 6 months	11,141	30.3	28.9	27.0
Feelings of unattachment	9,453	7.1	6.9	7.3
Parenting—Least warmth	11,389	23.8	25.2	24.9
Parenting—Least choice	11,246	32.3	33.6	32.8
Home learning environment	11,097	(10.3)	(10.4)	(10.1)
Maternal depression	11,820	10.1	8.6	10.4
Maternal smoking	11,835	22.9	19.3	25.2
Married, first marriage	9,535	71.5	73.9	68.9
Never married	9,535	10.8	9.6	13.4
Weekly family take-home income > £400	8,645	28.2	23.2	22.6
Weekly family take-home income < £100	8,645	7.8	6.6	10.1
Child sometimes ill or hardly ever well	11,018	5.0	4.9	5.3
Development score, 18 months	10,396	(38.0)	(37.9)	(37.8)
Behavioral difficulties, 47 months	9,457	(8.9)	(8.9)	(9.3)
Confounders of *M*-*Y* not caused by *C* or *X*				
Sex				
Female	13,976	48.3	48.9	49.0
Age at school entry assessment, < 55 m	9,476	51.3	50.6	51.1
Confounder of *X*-*M* or *X*-*Y*				
Parental ethnicity, non-White	12,337	4.9	2.7	4.7
Maternal age, years	13,978	(27.2)	(27.9)	(27.2)

Note

Poor educational achievement was defined as not achieving at least five General Certicate of Secondary Education at grade A^*^–C including English and Maths.

**Table 2:** Educational Achievement and Descriptive Characteristics by Socioeconomic Index and School Entry Assessment Score

	Socioeconomic Index		
	High % or *M* [95% CI]	Medium % or *M* [95% CI]	Low % or *M* [95% CI]
Poor educational achievement	22.4 [20.8, 24.0]	47.1 [45.8, 48.4]	73.6 [72.1, 75.1]
School entry assessment			
High	52.9 [50.4, 55.4]	31.9 [30.6, 33.2]	16.2 [14.8, 17.6]
Medium	37.3 [35.0, 39.5]	47.1 [45.7, 48.5]	44.8 [42.8, 46.9]
Low	9.8 [8.4, 11.3]	21.0 [19.8, 22.0]	39.0 [37.1, 40.8]
Birth weight, g	3,454.1 [3,433.2, 3,475.0]	3,397.3 [3,382.7, 3,411.8]	3,326.7 [3,307.1, 3,346.3]
Preterm birth, < 37 weeks	5.1 [4.2 6.0]	5.8 [5.2, 6.4]	6.2 [5.4, 7.0]
Breastfeeding, never	8.5 [7.4 9.7]	27.5 ([26.2, 28.7]	44.0 [41.9, 46.1]
Breastfeeding, at least 6 months	51.8 [49.7, 53.8)	22.7 [21.4, 23.9]	15.6 [14.2, 17.0]
Poor attachment	7.9 [6.8, 9.1)	6.1 [5.4, 6.9]	8.9 [7.3, 10.4]
Parenting—Least warmth	19.0 [17.5, 20.6)	26.0 [24.8, 27.2]	27.3 [25.6, 29.1]
Parenting—Least choice	30.4 [28.6, 32.3)	34.7 [33.4, 36.0]	31.3 [29.5, 33.2]
Home learning environment	10.5 [10.4, 10.5)	10.2 [10.1, 10.2]	9.7 [9.6, 9.7]
Maternal depression	8.0 [6.9–9.1)	8.0 [7.3, 8.8]	16.2 [14.8, 17.6]
Maternal smoking	9.5 [8.3–10.7)	19.3 [18.2, 20.4]	46.7 [44.8, 48.5]
Married, first marriage	79.8 [78.1, 81.4)	77.1 [75.7, 78.5]	47.2 [44.8, 49.7]
Never married	6.2 [5.2, 7.1)	6.9 [6.1, 7.7]	29.4 [27.0, 31.8]
Weekly family take-home income > £400	55.2 [53.1, 57.3)	19.1 [17.9, 20.2]	4.4 [3.5, 5.3]
Weekly family take-home income < £100	1.3 [0.9, 1.2)	5.0 [4.3, 5.7]	25.0 [23.0, 27.1]
Child sometimes ill or hardly ever well	4.2 [3.4, 5.1)	4.4 [3.8, 5.1]	7.5 [6.3, 8.6]
Development—Denver	37.8 [37.5, 38.0)	37.8 [37.6, 37.9]	37.9 [37.7, 38.2]
Behavior—SDQ	8.1 [7.9, 8.3)	9.1 [8.9, 9.2]	10.5 [10.3, 10.7]
Parental ethnicity, non-White	4.4 [3.6, 5.2)	2.7 [2.2, 3.1]	8.2 [7.1, 9.2]
Maternal age	30.2 [30.0, 30.3]	27.4 [27.2, 27.5]	24.6 [24.4, 24.8]

	School entry assessment		
	High % [95% CI]	Medium % [95% CI]	Low % [95% CI]
Poor educational achievement	24.2 [22.8, 25.7]	52.2 [50.8, 53.6]	78.2 [76.5, 80.0]

Note

Poor educational achievement was defined as not achieving at least five General Certicate of Secondary Education at grade A^*^–C including English and Maths. (*n* = 11,764). SDQ = Strengths and Difficulties Questionnaire.

Table3 shows the total effects model of low socioeconomic position (RR = 2.99, 95% CI [2.76, 3.23]) and low school entry scores (RR = 3.01, 95% CI [2.81, 3.21]) on risk of poor educational achievement. The marginal structural model (Model 2) using inverse probability weights resulted in children of medium and low socioeconomic position having 73% and 128% increased risk of poor educational achievement, respectively. Larger relative risks for socioeconomic position were observed with this marginal structural model than in the conventional regression (Model 3), although results were similar.

**Table 3:** Association of Socioeconomic Index and School Entry Assessment on Poor Educational Achievement at Ages 15–16

	Model 1, total effects		Model 2, controlled direct effects		Model 3, conventional regression	
	RR [95% CI]	*p*	RR [95% CI]	*p*	RR [95% CI]	*p*
Socioeconomic Index						
High	1.00		1.00		1.00	
Medium	2.00 [1.85, 2.17]	< .001	1.73 [1.54, 1.95]	< .001	1.53 [1.41, 1.66]	< .001
Low	2.99 [2.76, 3.23]	< .001	2.28 [2.01, 2.59]	< .001	1.77 [1.62, 1.93]	< .001
School entry assessment score						
High	1.00		1.00		1.00	
Medium	2.06 [1.93, 2.21]	< .001	1.71 [1.55, 1.89]	< .001	1.77 [1.66, 1.89]	< .001
Low	3.01 [2.81, 3.21]	< .001	2.22 [1.97, 2.50]	< .001	2.27 [2.11, 2.43]	< .001

Note

Model 1 is adjusted for confounders, *C* (parent ethnicity, maternal age) by inclusion of covariates in separate regression models for Socioeconomic Index, *X*, and school entry assessment score, *M*. Model 2 is a marginal structural model weighted for all confounders *C* (parent ethnicity, maternal age) and *L* (birth weight, preterm birth, breastfeeding, maternal feelings of unattachment, parenting, home learning environment, maternal depression, maternal smoking, marital status, income, child health, development and behavioral difficulties, sex, age at school entry assessment). Model 3 is the conventional regression model including *X*, *M*, and all confounders *C* and *L*. (*n* = 11,764). RR = relative risk.

Table4 shows the results of using the marginal structural model to simulate the intervention scenarios. In the universal intervention Scenario 1, where school entry scores of all children are increased, the predicted proportion of children with poor educational achievement was not changed. The mean school entry assessment score improved from 12.8 (*SD* = 3.2) to 13.5 (*SD* = 3.2) under Scenario 1 where the score was improved by 0.2 *SD* (equal to moving up 0.65 points). This improvement was therefore not large enough to move any individuals from low to medium, or from medium to high, in the categorical mediator that was used in the marginal structural model. The mean school entry assessment score increased to 14.0 (*SD* = 3.1), 13.9 (*SD* = 3.5), and 13.6 (*SD* = 3.1) under intervention Scenarios 2–4, respectively. When the school entry scores were improved more for socioeconomically disadvantaged children (intervention Scenario 2, Table4), the overall risk of poor educational achievement reduced by 4.5% and the excess risk experienced by the most disadvantaged children reduced by 15.7% from 47.0 per 100 to 39.6 per 100. The excess risk was calculated by subtracting the risk in the high (least disadvantaged) socioeconomic group from the risk in the more disadvantaged (medium or low) socioeconomic group. In the perverse universal intervention Scenario 3, where school entry scores were improved more for advantaged children, the predicted proportion with poor educational achievement reduced by 2.0% but the excess risk experienced by the low and medium socioeconomic groups, relative to the high socioeconomic group, increased by 9.8% and 19.4%, respectively. A similar reduction in absolute socioeconomic inequality comparing the least to the most disadvantaged children is seen for the progressive universal Scenario 2 and the proequity targeted Scenario 4 (15.7%). Increasing school entry scores only for socioeconomically disadvantaged children also reduced the overall predicted proportion with poor educational achievement by 4.5%. Relative socioeconomic inequality remained under all scenarios, but increased in the perverse universal Scenario 3 (RR = 3.67, for children of low Socioeconomic Index). Results using the nonimputed sample of respondents with complete data on all variables in the model (*n *=* *4,290, Table S6.2 in Appendix S6) show that effects of each of the hypothetical scenarios are consistent with the imputed analyses.

**Table 4:** Effects of Hypothetical Interventions to Improve School Entry Assessment Score on Predicted Risk of Poor Educational Achievement, Overall and by Socioeconomic Index

	Risk per 100 [95% CI]	Reduction in overall risk (%)	Excess risk (per 100)	Reduction in excess risk (%)	Crude relative risk [95% CI]
Baseline predicted risk of poor educational achievement					
Overall	49.3 (48.7–49.9)				
Socioeconomic Index					
High	23.9 [22.6, 25.2]		Ref		Ref
Medium	47.6 [46.8, 48.4]		23.7		1.99 [1.85, 2.14]
Low	70.9 [69.5, 72.4])		47.0		2.97 [2.76, 3.19]
Intervention Scenario 1 (universal): Increase school entry assessment score by 0.2 *SD* for all children					
Overall	49.3 [48.7, 49.9]	0.0			
Socioeconomic Index					
High	23.9 [22.6, 25.2]		Ref		Ref
Medium	47.6 [46.8, 48.4]		23.7	0.0	1.99 [1.85, 2.14]
Low	70.9 [69.5, 72.4]		47.0	0.0	2.97 [2.76, 3.19]
Intervention Scenario 2 (progressive universal): Increase school entry assessment score of children with low Socioeconomic Index by 0.8 *SD* and others by 0.2 *SD*					
Overall	47.1 [46.5, 47.7]	4.5			
Socioeconomic Index					
High	23.9 [22.6, 25.2]		Ref		Ref
Medium	47.6 [46.8, 48.4]		23.7	0.0	1.99 (1.85, 2.14]
Low	63.5 [61.9, 65.1]		39.6	15.7	2.66 (2.47, 2.86]
Intervention Scenario 3 (perverse universal): Increase school entry assessment score of children with high Socioeconomic Index by 0.8 *SD* and others by 0.2 *SD*					
Overall	48.3 [47.6, 48.9]	2.0			
Socioeconomic Index					
High	19.3 [18.6, 20.2]		Ref		Ref
Medium	47.6 [46.8, 48.4]		28.3	−19.4	2.47 [2.27, 2.68]
Low	70.9 [69.5, 72.4]		51.6	−9.8	3.67 [3.39, 3.99]
Intervention Scenario 4 (proequity targeted): Increase school entry assessment score by 0.8 *SD* only among children with low Socioeconomic Index					
Overall	47.1 [46.6, 48.2]	4.5			
Socioeconomic Index					
High	23.9 [22.6, 25.2]		Ref		Ref
Medium	47.6 [46.8, 48.4]		23.7	0.0	1.99 [1.85, 2.14]
Low	63.5 [61.9, 65.1]		39.6	15.7	2.66 [2.47, 2.86]

Note

*n* = 11,764.

## Discussion

Proequity interventions to improve school entry academic skills of disadvantaged children were estimated to reduce absolute socioeconomic inequality in poor educational achievement between the least and most disadvantaged groups by 15.7% for progressive universal or targeted interventions. Improving school entry academic skills of disadvantaged children was also estimated to reduce the proportion of children with poor educational outcomes at ages 15–16 by 4.5%. In 2012, there were approximately 621,000 pupils at the end of Key Stage 4 in England (Department for Education, 2013). Under Scenario 2, if we could reduce the proportion of children with poor educational achievement from 49.3% to 47.1%, 13,700 more children would be achieving a good educational outcome that is necessary for gaining access to further education, employment, and training in the United Kingdom.

Our results also demonstrate strong residual effects of socioeconomic disadvantage early in life that are not mediated by school entry academic skills. Children of low socioeconomic position are 2.3 times more likely than children of high socioeconomic position to have a poor educational outcome at ages 15–16. Improving early life socioeconomic disadvantage is likely to improve educational outcomes at ages 15–16, but intervening to address the fundamental conditions that limit life prospects of children, including poverty, has proven to be complex (Shaw, Smith, & Dorling, 2005; Shonkoff et al., 2012).

The majority (78%) of children with a low school entry assessment score in this study had poor educational achievement at ages 15–16. Poorer school entry academic skills may be difficult to compensate for later in life because early childhood is a particularly sensitive period for brain formation (Heckman, 2006; Naudeau, Kataoka, Valerio, Neuman, & Kennedy Elder, 2011). While interventions to improve educational achievement may need to occur throughout schooling, early childhood interventions have been shown to have a higher rate of return per investment than interventions targeting older children or adults (Heckman, 2008; Heckman, Stixrud, & Urzua, 2006), and protecting young children from social and economic adversity is likely to have a positive influence on many contemporary problems including low educational achievement, diminished economic productivity, criminality, and health inequalities (Committee on Psychosocial Aspects of Child Family Health et al., 2012; Heckman, 2008). Previous research has also indicated that children of well-educated or wealthy parents, even if they do poorly at school entry, are more likely to do well in later school education than children of poor parents or parents with a low level of education (Feinstein, 2003; The Marmot Review, 2010), and while many children from disadvantaged backgrounds gain advantage via school readiness, children from more advantaged families gain even more (Bynner et al., 2000). This points to the influence of “noncognitive” or personality characteristics, including social expectations and aspects of self-regulation like persistence, motivation, and ability to concentrate that may be intergenerationally transmitted (Heckman & Rubinstein, 2001).

We have examined effects of interventions on academic achievement that are consistent with current evidence from well-designed randomized and quasi-experimental studies (Duncan & Magnuson, 2013). Nevertheless, the proequity scenarios were based on an effect of 0.8 *SD*, which is toward the upper end of what has been observed in controlled trials. Whether it is possible to achieve such effects in scaled-up interventions within normal practice is not clear, although a recent study estimated intervention effects on low-income children to be 0.82 *SD* larger than high-income children (Duncan & Sojourner, 2013). The relatively weak intervention effects shown in Scenario 1 of the order of 0.2 *SD* do not appear to have an effect on population levels or social inequalities in educational achievement in these data. This highlights the ongoing need to develop more powerful single interventions and interventions that are better integrated across ages and provide compound effects from birth to age 5 to increase the magnitude of their positive influences on educational outcomes. There is also a case for early intervention to be followed up later in childhood and adolescence so that the effects of early investment are not lost (Cunha & Heckman, 2007), although effects of school interventions are mixed (Clifton & Cook, 2012; Heckman, 1999; Holmlund & Silva, 2009; Keogh, Bond, & Flaxman, 2006).

While the highest proportion of children with low school entry assessment scores (39.0%) is seen among the lowest socioeconomic group, the burden is similar among the medium socioeconomic group because although a smaller proportion of the medium group have low school entry assessment scores (21.0%), they make up a larger share of the population (48.6% vs. 29.5%). This may partly explain why only a 4.5% reduction in risk of poor educational achievement in the overall population at ages 15–16 is observed as a result of interventions that focus on improving school entry academic skills of only socioeconomically disadvantaged children. Targeted interventions for vulnerable groups (Scenario 4) have the potential to reduce the difference in educational achievement between the low - and high-socioeconomic groups by 16%, but a similar reduction in inequalities and the greatest reduction in overall population burden of poor educational achievement (5.4%) is seen in Scenario 2, which reflects the progressive universalism principle in providing universal services for all children, with more intensive support programs that have a greater effect on school readiness for children with greater need (Lynch et al., 2010). Providing services under a progressive universal approach has implications for policy and practice. In Scotland, for example, increased service provision for vulnerable children means that the number of visits from a health visitor offered universally to children is reduced (Wood, Stockton, & Brown, 2013). Determining eligibility criteria for targeted programs to improve school readiness may require consideration of factors in addition to socioeconomic position (Chittleborough, Lawlor, & Lynch, 2011).

This research required a cohort with richly characterized data from pregnancy to age 16 and benefited from the large, relatively representative sample and the longitudinal design of ALSPAC, with linkage to objective measures of educational achievement in the NPD. A strength of this study was the ability to include a large number of confounding variables, including maternal smoking and depression (Leftwich & Collins, 1994), breastfeeding (Kramer et al., 2008), and home environment and parenting (Dearden, Sibieta, & Sylva, 2011). Parental aspirations and attitudes to education that may help explain socioeconomic inequalities in educational achievement (Gregg & Washbrook, 2011) were not measured in ALSPAC before school entry. An advantage of marginal structural models over conventional methods is that we can better account for confounders of the mediator–outcome association. The estimated effects rely on the assumption that there is no unmeasured confounding. The sensitivity analyses we conducted (Appendix S4) demonstrate that unmeasured confounding is unlikely to account for the entire causal effect observed or the relative differences between the intervention scenarios.

The use of a teacher-assessed measure of school entry academic skills may have validity limitations (Harlen, 2004), but this was the assessment used by local educational authorities. Our use of categorical exposure and mediator variables to simplify the interpretation of the marginal structural models may have resulted in a loss of information from the continuous variables (DeCoster, Iselin, & Gallucci, 2009). School entry academic skills in this study assessed both early literacy and maths, meaning that we cannot disentangle the separate effects of literacy and maths on later academic achievement. There is a need for reliability testing of GCSE scoring (He, 2010), although the GCSE outcome measure used in this study is widely recognized in the United Kingdom as being a requirement for further study and higher quality employment opportunities (Unwin, 2010; Wolf, 2011).

The results in this study should be seen as contextualized, and transferability to other populations would depend on several assumptions. These assumptions include the effects from the randomized trials being universally applicable, as an effect size of 0.8 *SD* is toward the upper range of observed effects and may not be achievable in all settings outside of efficacy trials. In addition, transferability of results also depends on the controlled direct effect of socioeconomic disadvantage on educational achievement, the association of confounders with the mediator and outcome, and the distributions of socioeconomic disadvantage and school entry academic skills being similar across populations. Cross-country cohort comparisons are required to test these assumptions. The similarity of our results to those of Duncan and Sojourner (2013) in the United States, where patterns of socioeconomic inequalities and social policy may differ from the United Kingdom, may offer some support for generalizability of our findings but this needs to be tested in other populations.

While socioeconomic disadvantage in early childhood has a strong, enduring effect on later educational outcomes, in these data, proequity progressive universal early childhood interventions (effect sizes 0.2 *SD* for universal and 0.8 *SD* for proequity) to improve school entry academic skills, are estimated to reduce socioeconomic inequality in poor educational outcomes at ages 15–16 by about 15% while also improving by 5% overall population levels of academic achievement.

## Supporting Information

Appendix S1 Selected Early Childhood Interventions Shown to Improve School Entry Academic Skills **Appendix S2** Additional Information About Marginal Structural Models and Conventional Regression Approaches for Assessing Direct and Indirect Effects of *X* on *Y* Via *M* **Appendix S3** Association of Socioeconomic Variables With Outcome (Educational Achievement) and Mediator (School Entry Academic Skills) **Appendix S4** Sensitivity Analyses to Assess Effect of Unmeasured Confounding **Appendix S5** Multiple Imputation Method **Appendix S6** Complete Case Analyses **Appendix S7** Sample Sizes Included for Analyses
