# Supplementary material for: Effects of Simulated Interventions to Improve School Entry Academic Skills on Socioeconomic Inequalities in Educational Achievement
Source: Child Dev. 2014 Oct 18;85(6):2247–62. doi: 10.1111/cdev.12309 (PMC4257067; doi:10.1111/cdev.12309)
Supplement: Appendix S1 — Selected Early Childhood Interventions Shown to Improve School Entry Academic Skills Appendix S2 Additional Information About Marginal Structural Models and Conventional Regression Approaches for Assessing Direct and Indirect Effects of X on Y Via M Appendix S3 Association of Socioeconomic Variables With Outcome (Educational Achievement) and Mediator (School Entry Academic Skills) Appendix S4 Sensitivity Analyses to Assess Effect of Unmeasured Confounding Appendix S5 Multiple Imputation Method Appendix S6 Complete Case Analyses Appendix S7 Sample Sizes Included for Analyses [file cdev0085-2247-SD1.docx]

Effects of simulated interventions to improve school entry academic skills on socioeconomic inequalities in educational achievement

**Appendix S1**

**Selected early childhood interventions shown to improve school entry academic skills**

The largest effects on development have been demonstrated in randomised trials of the Abecedarian and High Scope/Perry Preschool programs, which were high quality child care and preschool interventions for low income African American children ([Currie, 2001](#_ENREF_8)). The Abecedarian program had positive effects on cognitive ability at age four ([Campbell, Pungello, Miller-Johnson, Burchinal, & Ramey, 2001](#_ENREF_4)). Effects of preschool treatment on reading and maths skills persisted into adulthood, with higher scores on intellectual and academic measures at age 21 ([Campbell, Ramey, Pungello, Sparling, & Miller-Johnson, 2002](#_ENREF_5)). This program also had long-term health effects with better cardiovascular risk factor outcomes observed for participants in their mid-30s ([Campbell et al., 2014](#_ENREF_3)). The High Scope/Perry Preschool program translated into better high school graduation rates, employment rates, higher earnings, and lower crime rates at age 40 ([Schweinhart et al., 2005](#_ENREF_28)). The Chicago School Readiness Project, a cluster randomised efficacy trial implemented in Head Start preschool classrooms, showed that an intervention targeting behavioural self-regulation improved low income children’s literacy and math skills ([Raver et al., 2011](#_ENREF_23)). Another randomised controlled trial demonstrating improved cognitive development of children at age six involved nurse home visits to economically disadvantaged first time mothers from pregnancy until the child was two years old ([Olds et al., 2004](#_ENREF_21)).

Using a quasi-experimental approach, a high quality preschool program in five US states was shown to have positive effects on vocabulary, maths and print awareness at school entry ([Wong, Cook, Barnett, & Jung, 2008](#_ENREF_34)). Non-experimental data from the Head Start program, a public preschool program offered to children aged three to five years in low income families ([Barnett, 1995](#_ENREF_1); [Karoly et al., 1998](#_ENREF_17)), has shown that Head Start had positive effects on vocabulary test scores ([Currie & Thomas, 1995](#_ENREF_9)). These effects faded out for African American children, most likely a reflection of the differential experiences of African Americans and white children after they finish the Head Start program ([Currie & Thomas, 2000](#_ENREF_10)). By the time they were in their early-twenties, white Head Start participants were more likely to have completed high school and attended college, and African American participants were less likely to have been booked or charged with a crime relative to their siblings who did not attend Head Start ([Garces, Thomas, & Currie, 2002](#_ENREF_15)). Additionally, observational studies, including the Effective Provision of Pre-School Education project and the National Institute of Child Health and Human Development Study, demonstrated that centre-based child care and preschool were associated with improved language development, early number concepts and pre-reading at school entry ([NICHD Early Child Care Research Network, 2002](#_ENREF_20); [Sammons et al., 2004](#_ENREF_27)). Systematic reviews have also indicated that preschool interventions have some longer term benefits for health-related outcomes ([D'Onise, Lynch, Sawyer, & McDermott, 2010](#_ENREF_11); [D'Onise, McDermott, & Lynch, 2010](#_ENREF_12)).

A summary of these interventions, and their effects on various measures of school entry academic skills is included in Table S1.1.

Table S1.1

*Summary of selected early childhood interventions shown to improve school entry academic skills by approximately age 5 years*

| **Intervention** | **Description** | **Results (effect sizes shown in brackets)** |
| --- | --- | --- |
| Abecedarian ([Campbell et al., 2001](#_ENREF_4)) | Prospective randomized trial in which high-quality child care was randomly assigned to low-income African-American children (*n* = 111, 57 treatment, 54 control) | Cognitive ability assessed using the Stanford-Binet Intelligence Scale at age 4 (0.74) |
| High Scope/Perry Preschool ([Karoly, Kilburn, & Cannon, 2005](#_ENREF_18)) | Randomised experiment of an intensive, high quality pre-kindergarten education program delivered to children born in poverty (*n* = 58 treated, 65 control). | Cognitive ability assessed using the Stanford-Binet Intelligence Scale at age 5 (0.97) |
| Chicago School Readiness Project ([Raver et al., 2011](#_ENREF_23)) | Cluster-randomized efficacy trial implemented in 35 Head Start–funded classrooms (*n* = 602 children). This project was designed to support low-income children’s self-regulation and their opportunities to learn in early educational settings. | Shortened version of the Peabody Picture Vocabulary Test (PPVT-III, 24 questions) (0.34)  Letter naming of the letters of the alphabet divided into three groups (0.63)  Early math skills included basic addition and subtraction (0.54) |
| Nurse Family Partnership ([Olds et al., 2004](#_ENREF_21)) | Randomised controlled trial of nurse home visits to economically disadvantaged first time mothers in Memphis, Tennessee. During pregnancy 1139 women were randomised, and assessments at age 6 were completed for 615children. | Receptive vocabulary assessed using the Peabody Picture Vocabulary Test (PPVT-III) at age 6 (0.17) |
| Effective Provision of Pre-school Education ([Sammons et al., 2004](#_ENREF_27)) | Longitudinal study of children (*n* = 2857) attending 141 preschools in England of different types (vs. not attending preschool) | British Ability Scales Second Edition (BASII) at primary school entry:  Language development (0.44)  Early number concepts (0.44)  Pre-reading (0.28) |
| Early Childhood Longitudinal Study-Kindergarten Class ([Magnuson, Ruhm, & Waldfogel, 2007](#_ENREF_19)) | Longitudinal study of children (*n* = 10,224) compared effects of attending prekindergarten with attending other centre-based care, other non-parental care, or exclusively parental care. Results here are for prekindergarten vs. exclusively parental care. | Reading and Math skills assessments developed for this study and assessed at kindergarten (school entry, 5.7 years)  Reading (0.18)  Maths (0.17)  Effects were stronger among socioeconomically disadvantaged. |
| National Institute of Child Health & Human Development (NICHD) longitudinal study ([NICHD Early Child Care Research Network, 2002](#_ENREF_20)) | Longitudinal study from birth to age 4.5 years (*n* = 1364). Routinely experienced centre-based care and pre-Kindergarten. Compared children whose child care was in the highest third of quality with children whose child care quality was in the bottom third. | Language competence assessed using the Preschool Language Scale at age 4.5 years (0.15)  Pre-academic skills assessed as a composite score from two subtests (Letter-Word Identification, Applied Problems) of the Woodcock Johnson Achievement and Cognitive Batteries (0.24) |
| Pre-Kindergarten in five US states ([Wong et al., 2008](#_ENREF_34)) | High quality pre-kindergarten program with qualified, well paid teachers, staff to child ratio 1:10 and maximum group size of 20. Quasi-experimental design. Treatment groups consisted of children who had completed pre-kindergarten in spring 2004 and were starting kindergarten in fall 2004. Comparison children were 4-year-olds just starting pre-K in fall 2004. Sample sizes in each state ranged from 720 to 2072. | Assessments were at the end of pre-kindergarten at age 5 years.  Receptive vocabulary assessed using the Peabody Picture Vocabulary Test (PPVT-III):  New Jersey (0.36)  Oklahoma (0.28)  Mathematical skills were measured with the Woodcock-Johnson Tests of Achievement, 3rd Edition:  Michigan (0.53)  New Jersey (0.23)  Print Awareness abilities were measured using the print awareness subtest of the Preschool Comprehensive Test of Phonological and Print Processing (Pre-CTOPP):  Michigan (1.09)  New Jersey (0.32)  South Carolina (0.78)  West Virginia (0.92) |
| Pre-Kindergarten in Oklahoma ([Gormley, Gayer, Phillips, & Dawson, 2005](#_ENREF_16)) | High quality universal pre-kindergarten program with qualified, well paid teachers, staff to child ratio 1:10 and maximum group size of 20. The treatment group consisted of kindergarten children who were enrolled in the pre-kindergarten program the previous year (*n* = 3149). The control group consisted of children who had just begun pre-kindergarten at the time of testing (*n* = 1567). | Three subtests of the Woodcock–Johnson Achievement Test were used at age 5:  Letter–Word Identification (0.79)  Spelling (0.64)  Applied Problems (0.38) |
| Head Start ([Deming, 2009](#_ENREF_14)) | Head Start provides preschool education for low income children and additional services such as such as medical, dental, and mental health care, nutrition and child development assistance and education for parents. Data from the National Longitudinal Mother-Child Supplement (CNLSY), which surveyed the mothers of the National Longitudinal Survey of Youth (NLSY) 1979 every two years from 1986 until 2004 (*n* = 3698). | Summary index of test scores that included standardized Peabody Picture Vocabulary Test and the Peabody Individual Achievement Math and Reading Recognition subtests at ages 5-6 (0.15) |

**Appendix S2**

**Additional information about marginal structural models and conventional regression approaches for assessing direct and indirect effects of *X* on *Y* via *M***

The causal diagram for our study is depicted in Figure S2.1. The effect of socioeconomic disadvantage in early life *X*, has a direct effect on educational achievement *Y*, (pathway 1), and an effect mediated by school entry academic skills *M* (pathways 2 and 3). School entry academic skills also have an independent effect on educational achievement (pathway 3). The effect of socioeconomic disadvantage on educational achievement will also be mediated by other factors such as birth characteristics, parenting, and home environment (pathway 4-5), all of which may be confounders *L*, of *M*−*Y* (pathway 3).

10

1

9

3

2

*Educational achievement*

*Y*

*School entry academic
skills, M*

*Socioeconomic disadvantage*

*X*

8

*C*

6

4

5

7

*L*

Pregnancy

Age 5

Age 16

Birth

Figure S2.1

*Causal diagram of the hypothesized effects of socioeconomic disadvantage during pregnancy (X) and school entry academic skills at age 5 (M) on poor educational achievement at age 15-16 (Y)*

*Note*. *C* represents confounders of the association between *X*, *M* and *Y* (parental ethnicity, maternal age). *L* represents confounders of the effect of school entry academic skills, *M*, on poor educational achievement, *Y*, measured between birth and age 5 (birth weight, preterm birth, breastfeeding, maternal feelings of unattachment, parenting, home learning environment, maternal depression, maternal smoking, marital status, income, child health, development and behavioural difficulties, sex, age at school entry assessment).

The conventional approach ([Baron & Kenny, 1986](#_ENREF_2)) to assessing direct effects of *X* on *Y* and indirect effects of X on *Y* via *M* is to fit a regression model of *Y* conditional on *X* and any confounders of the *X-Y* association, and then examine how the coefficient of *X* changes when the mediator, *M*, is added to the model. The difference between the two coefficients of *X* is a measure of the reduction in the total effect of *X* that is mediated through *M*. Structural equation modelling has also been used to estimate total, direct and indirect effects but this technique is restricted to situations where all associations are linear ([Pearl, 2001](#_ENREF_22)), and is not recommended for causal mediation analyses ([VanderWeele, 2012](#_ENREF_31)). Most importantly, recent advances in causal inference ([Daniel, De Stavola, & Cousens, 2011](#_ENREF_13); [VanderWeele & Vansteelandt, 2009](#_ENREF_32)) show that the conventional method is invalid when confounders, *L*, of the *M-Y* association exist because conditioning on *M* induces an association between *X* and *L*, opening up a backdoor path *X*–*L*–*Y* (pathway 4-5 in Figure 1). Including *L* as a covariate in the regression does not solve this problem arising from conditioning on *M* when, as in our case, *L* is a consequence of *X*. If we condition on *L* we block the backdoor path *X*–*L*–*Y*, which also blocks part of the direct effect of *X* on *Y* that is not mediated through *M* ([Daniel et al., 2011](#_ENREF_13)). Conditioning on *L* could also introduce collider bias because L is a common effect of the causes of exposure and outcome ([Cole et al., 2010](#_ENREF_7)). To overcome these limitations we estimate the controlled direct effect of socioeconomic disadvantage on poor educational achievement using marginal structural models. The controlled direct effect in this case is the effect of socioeconomic disadvantage on educational achievement if school entry academic skills were controlled at some specified level uniformly in the population ([Pearl, 2001](#_ENREF_22); [Robins & Greenland, 1992](#_ENREF_24); [VanderWeele & Vansteelandt, 2009](#_ENREF_32)). Marginal structural models are relatively new in the field of epidemiology and are applied to observational data to better estimate the causal effect of an exposure, taking into account covariates that may be both confounders and intermediate variables ([Robins, Hernan, & Brumback, 2000](#_ENREF_25)). A marginal structural model differs from regression because the model is for counterfactual outcomes rather than observed outcomes, and it is a marginal model, not conditional on any covariates ([VanderWeele, 2009](#_ENREF_29)). In the counterfactual, or potential outcomes, framework, each individual has a potential outcome for each possible treatment, or exposure, condition ([Coffman & Zhong, 2012](#_ENREF_6)). In a marginal structural model, covariates are removed from the model but accounted for by weighting each participant by the inverse of the probability of them being exposed as a function of their covariates. The weighting creates a pseudopopulation in which the association of confounders with exposure is removed ([Robins et al., 2000](#_ENREF_25)). To estimate an unbiased causal effect we assume that there is no unmeasured confounding ([Robins et al., 2000](#_ENREF_25)). This assumption is not unique to marginal structural models, it is also applied to conventional regression and structural equation models. An advantage of marginal structural models over conventional methods is that we can better account for the confounders, *L*, of the mediator – outcome association.

**Appendix S3**

**Association of socioeconomic variables with outcome (educational achievement) and mediator (school entry academic skills)**

Table S3.1

*Association of individual socioeconomic variables included in the socioeconomic index with poor educational achievement and low school entry academic skills*

|  | **Poor educational achievement** | **School entry academic skills** |
| --- | --- | --- |
|  | RR (95% CI) | RR (95% CI) |
| *Parents’ highest education level* | *n* = 10525 | *n* = 8387 |
| Degree or higher | 1.00 | 1.00 |
| A level | 1.97 (1.78-2.18) | 1.96 (1.62-2.37) |
| O level | 2.72 (2.47-3.00) | 2.64 (2.19-3.19) |
| Less than O level | 3.73 (3.40-4.10) | 4.37 (3.64-5.24) |
| *Parents’ social class* | *n* = 9740 | *n* = 7726 |
| I or II | 1.00 | 1.00 |
| III | 1.63 (1.52-1.75) | 1.69 (1.47-1.94) |
| IV or V | 2.24 (2.08-2.42) | 2.40 (2.07-2.78) |
| *Home ownership* | *n* = 11002 | *n* = 8805 |
| Owned or mortgaged | 1.00 | 1.00 |
| Rented or other | 1.73 (1.67-1.80) | 1.99 (1.85-2.13) |
| *Household crowding* | *n* = 10816 | *n* = 8640 |
| .5 or less persons per room | 1.00 | 1.00 |
| > .5 to .75 persons per room | 1.32 (1.25-1.39) | 1.48 (1.34-1.64) |
| > .75 to 1 persons per room | 1.73 (1.64-1.82) | 2.04 (1.84-2.26) |
| > 1 person per room | 2.00 (1.88-2.12) | 2.65 (2.35-2.99) |
| *Parents’ employment status* | *n* = 10568 | *n* = 8425 |
| Employed | 1.00 | 1.00 |
| Unemployed or seeking job | 1.42 (1.35-1.49) | 1.55 (1.40-1.71) |
| *Financial difficulties score* | *n* = 10219 | *n* = 8143 |
| 0 to 8 | 1.00 | 1.00 |
| > 8 | 1.42 (1.35-1.49) | 1.55 (1.40-1.71) |

*Note*. Poor educational achievement was defined as not achieving at least 5 GCSE at grade A*-C including English and Maths.

**Appendix S4**

**Sensitivity analyses to assess effect of unmeasured confounding**

Using a marginal structural model in this observational study to determine the causal effect of socioeconomic disadvantage on poor educational achievement assumes no unmeasured confounding. We conducted sensitivity analyses to assess the effect of *U*, an unmeasured common cause of *X* and *Y*, on the controlled direct effect of socioeconomic disadvantage on poor educational achievement. The bias for the conditional controlled direct effect is defined as ([VanderWeele, 2010](#_ENREF_30))

$$Bias\left( {CDE}_{x,x*|c}^{RR}\left( m \right) \right)= \frac{1+\left( \gamma-1 \right)P(U=1|x,m,c)}{1+\left( \gamma-1 \right)P(U=1|x*,m,c)}$$

where $\gamma$is the direct effect of *U* on *Y*, and $P(U=1|x,m,c)$ is the prevalence of the unmeasured confounder for exposure levels *x* and *x**. *U* could be a single confounder, or it could be multiple confounding variables, but we assume that *U* is not correlated with *C*. The bias for a range of $\gamma$ and prevalence of the confounder is listed in Table S4.1.

The bias of *U* would be sufficient to completely explain the observed controlled direct effect (RR = 2.28 for children with low socioeconomic index and RR = 1.73 for children with medium socioeconomic index, see Table 3) only if *U* increased the risk of poor educational achievement three-fold ($\gamma=3$ in Table S4.1) and there was a difference in the prevalence of the confounder across socioeconomic groups of 80% (for example 90% for exposure level *x*, and 10% for exposure level *x**, Table S4.1). Unmeasured confounding may be present, but it is difficult to think of a variable in this context with such characteristics. As a comparison, in this study the association between parental non-white ethnicity and poor educational achievement was much smaller (RR = 1.12, 95% CI 1.03-1.22), and the prevalence of parental non-white ethnicity was 4.4% and 8.2% among children with high and low socioeconomic index, respectively. Our estimate of mediation of this effect by school entry academic skills may be biased by unmeasured confounders, though we have included an extensive range of plausible confounders in *L*. Furthermore, our main aim in this mediation analysis is to apply different plausible scenarios from intervention studies (where confounding is unlikely) and the ‘addition’ of these effects to our observed mediation would show the same relative effect of the interventions even if we had a biased estimate of mediation in our observational study.

Table S4.1

*Results of sensitivity analyses for the controlled direct effect of socioeconomic disadvantage*

| $\boldsymbol{\gamma}$ | $\boldsymbol{P(U=1\vert x,m,c)}$ | $\boldsymbol{P(U=1\vert}\boldsymbol{x}^{\boldsymbol{*}}\boldsymbol{,m,c)}$ | **Bias** |
| --- | --- | --- | --- |
| 1.5 | 0.5 | 0.5 | 1.00 |
| 1.5 | 0.6 | 0.4 | 1.08 |
| 1.5 | 0.7 | 0.3 | 1.17 |
| 1.5 | 0.8 | 0.2 | 1.27 |
| 1.5 | 0.9 | 0.1 | 1.38 |
| 2 | 0.5 | 0.5 | 1.00 |
| 2 | 0.6 | 0.4 | 1.14 |
| 2 | 0.7 | 0.3 | 1.31 |
| 2 | 0.8 | 0.2 | 1.50 |
| 2 | 0.9 | 0.1 | 1.73 |
| 2.5 | 0.5 | 0.5 | 1.00 |
| 2.5 | 0.6 | 0.4 | 1.19 |
| 2.5 | 0.7 | 0.3 | 1.41 |
| 2.5 | 0.8 | 0.2 | 1.69 |
| 2.5 | 0.9 | 0.1 | 2.04 |
| 3 | 0.5 | 0.5 | 1.00 |
| 3 | 0.6 | 0.4 | 1.22 |
| 3 | 0.7 | 0.3 | 1.50 |
| 3 | 0.8 | 0.2 | 1.86 |
| 3 | 0.9 | 0.1 | 2.33 |

*Note*. $\gamma$ = relative risk of the direct effect of *U* on *Y*; $P(U=1|x,m,c)$ = prevalence of the unmeasured confounder for exposure level *x*; $P(U=1|x^{*},m,c)$ = prevalence of the unmeasured confounder for exposure level *x**.

**Appendix S5**

**Multiple imputation method**

Multiple imputation by chained equation was used to impute missing data using the ‘mi impute chained’ command in Stata version 12.0. Missing data were imputed on the outcome, exposure, mediator and confounding variables for respondents who were alive at one year (*n* = 13978). The imputation model included all outcome, exposure, mediator and confounding variables as well as other predictors of ‘missingness’ – smoking and alcohol consumption during the first three months of pregnancy, maternal EPDS score at 18 weeks gestation, and number of siblings when the child was 4 years of age. A range of regression models was used in the imputation (regress, mlogit, ologit, logit, pmm, truncreg), depending on the type of variable being imputed. We generated 20 data sets with 50 iterations for each imputed data set. These iterations allow for the imputation process to converge, with the results of only the 50^th^ iteration being saved for each of the 20 imputed datasets requested. The results were obtained by averaging across the results from each of these 20 datasets using Rubin’s rules and the procedure takes account of the uncertainty produced by the imputation in calculating the correct standard error ([Royston, 2004](#_ENREF_26)). We used the multiple imputation then deletion technique ([Von Hippel, 2007](#_ENREF_33)) where analyses were conducted on respondents only with non-imputed outcome data (*n* = 11764). Imputing outcomes is necessary during the imputation process for the imputation of exposures and mediators but adds no further information for estimating the causal effect.

**Appendix S6**

**Complete-case analyses**

Table S6.1

*Complete-case analysis of educational achievement and descriptive characteristics by socioeconomic index and school entry assessment score (n = 4290)*

|  | **Socioeconomic Index** | | | | |
| --- | --- | --- | --- | --- | --- |
|  | **High** | | **Medium** | | **Low** |
|  | % or Mean  (95% CI) | | % or Mean  (95% CI) | | % or Mean  (95% CI) |
| Poor educational achievement | 19.4 (17.0-21.8) | | 42.2 (40.2-44.2) | | 67.7 (64.6-70.8) |
| School Entry Assessment |  | |  | |  |
| High | 54.5 (51.4-57.5) | | 35.1 (33.2-37.0) | | 22.7 (20.0-25.6) |
| Medium | 35.8 (32.9-38.7) | | 47.4 (45.4-49.4) | | 45.7 (42.4-49.0) |
| Low | 9.8 (8.1-11.8) | | 17.6 (16.1-19.2) | | 31.7 (28.7-34.9) |
| Birth weight, g | 3461.9 (509.6) | | 3423.0 (535.4) | | 3426.8 (544.4) |
| Preterm birth, < 37 weeks | 4.3 (3.0-5.5) | | 4.8 (4.0-5.7) | | 4.5 (3.1-5.8) |
| Breastfeeding, never | 8.6 (7.1-10.5) | | 24.8 (23.1-26.6) | | 41.0 (37.7-44.3) |
| Breastfeeding, at least 6 months | 48.6 (45.6-51.7) | | 24.3 (22.6-26.1) | | 18.1 (15.7-20.8) |
| Poor attachment | 7.4 (5.8-9.0) | | 6.5 (5.6-7.5) | | 7.4 (5.7-9.2) |
| Parenting – least warmth | 19.6 (17.3-22.1) | | 26.7 (24.9-28.5) | | 27.9 (25.0-31.0) |
| Parenting – least choice | 30.3 (27.6-33.2) | | 35.4 (33.5-37.4) | | 32.5 (29.5-35.7) |
| Home learning environment | 10.6 (1.4) | | 10.4 (1.6) | | 10.0 (1.7) |
| Maternal depression | 7.8 (6.1-9.4) | | 6.7 (5.7-7.7) | | 14.8 (12.4-17.1) |
| Maternal smoking | 7.5 (5.9-9.2) | | 17.2 (15.6-18.7) | | 39.2 (36.0-42.5) |
| Married, first marriage | 81.7 (79.2-83.9) | | 78.6 (76.9-80.2) | | 51.9 (48.6-55.3) |
| Never married | 5.7 (4.5-7.3) | | 5.8 (4.9-6.8) | | 24.5 (21.7-27.5) |
| Weekly family take home income > £400 | 51.8 (48.8-54.9) | | 17.5 (16.0-19.1) | | 5.0 (3.8-6.7) |
| Weekly family take home income < £100 | 1.6 (1.0-2.6) | | 4.2 (3.5-5.1) | | 19.1 (16.6-21.9) |
| Child sometimes ill or hardly ever well | 3.6 (2.4-4.7) | | 4.8 (3.9-5.6) | | 6.8 (5.1-8.4) |
| Development - Denver | 37.8 (5.6) | | 37.8 (5.5) | | 38.4 (5.9) |
| Behaviour – SDQ | 8.1 (4.2) | | 8.8 (4.3) | | 10.2 (4.9) |
| Parental ethnicity, non-white | 3.1 (2.0-4.2) | | 1.7 (1.2-2.2) | | 4.7 (3.3-6.1) |
| Maternal age | 30.1 (3.8) | | 27.8 (4.0) | | 25.7 (5.0) |
|  | **School entry assessment** | | | | |
|  | **High** | **Medium** | | **Low** | |
|  | % (95% CI) | % (95% CI) | | % (95% CI) | |
| Poor educational achievement | 22.2 (20.2-24.3) | 45.2 (43.0-47.5) | | 73.4 (70.3-76.5) | |

*Note*. Poor educational achievement was defined as not achieving at least 5 GCSE at grade A*-C including English and Maths.

Table S6.2

*Complete-case analysis of association of socioeconomic index and school entry assessment on poor educational achievement at age 15-16 (n = 4290)*

|  | **Model 1, total effects** | | **Model 2,  controlled direct effects** | | **Model 3,  conventional regression** | |
| --- | --- | --- | --- | --- | --- | --- |
|  | RR (95% CI) | *p* value | RR (95% CI) | *p* value | RR (95% CI) | *p* value |
| Socioeconomic Index | |  |  |  |  |  |
| High | 1.00 |  | 1.00 |  | 1.00 |  |
| Medium | 2.11 (1.84-2.41) | < .001 | 1.87 (1.60-2.18) | < .001 | 1.64 (1.43-1.88) | < .001 |
| Low | 3.29 (2.86-3.77) | < .001 | 2.61 (2.20-3.09) | < .001 | 1.97 (1.70-2.29) | < .001 |
| School Entry Assessment score | |  |  |  |  |  |
| High | 1.00 |  | 1.00 |  | 1.00 |  |
| Medium | 1.99 (1.80-2.21) | < .001 | 1.69 (1.49-1.93) | < .001 | 1.73 (1.56-1.92) | < .001 |
| Low | 3.17 (2.86-3.51) | < .001 | 2.54 (2.22-2.91) | < .001 | 2.42 (2.17-2.70) | < .001 |

*Note*. RR = relative risk. Model 1 is adjusted for confounders, *C* (parent ethnicity, maternal age) by inclusion of covariates in separate regression models for Socioeconomic Index, *X* and School Entry Assessment score, *M*. Model 2 is a marginal structural model weighted for all confounders *C* (parent ethnicity, maternal age) and *L* (birth weight, preterm birth, breastfeeding, maternal feelings of unattachment, parenting, home learning environment, maternal depression, maternal smoking, marital status, income, child health, development and behavioural difficulties, sex, age at school entry assessment). Model 3 is the conventional regression model including *X*, *M*, and all confounders *C* and *L*.

Table S6.3

*Complete-case analysis of effects of hypothetical interventions to improve school entry assessment score on predicted risk of poor educational achievement, overall and by socioeconomic index (n = 4290)*

|  | **Risk per 100 (95% CI)** | **Reduction in overall risk** | **Excess risk (per 100)** | **Reduction in excess risk** | **Crude relative risk (95% CI)** |
| --- | --- | --- | --- | --- | --- |
| **Baseline predicted risk of poor educational achievement** | | | |  |  |
| Overall | 41.6 (41.0-42.2) |  |  |  |  |
| *Socioeconomic Index* | |  |  |  |  |
| High | 19.7 (19.0-20.4) |  | ref |  | ref |
| Medium | 41.9 (41.3-42.5) |  | 22.2 |  | 2.13 (1.86-2.43) |
| Low | 66.7 (65.7-67.6) |  | 47.0 |  | 3.39 (2.97-3.86) |
| **Intervention Scenario 1 (Universal):** Increase school entry assessment score by 0.2 SD for all children | | | | | |
| Overall | 41.6 (41.0-42.2) | 0.0% |  |  |  |
| *Socioeconomic Index* | |  |  |  |  |
| High | 19.7 (19.0-20.4) |  | ref |  | ref |
| Medium | 41.9 (41.3-42.5) |  | 22.2 | 0.0% | 2.13 (1.86- 2.43) |
| Low | 66.7 (65.7-67.6) |  | 47.0 | 0.0% | 3.39 (2.97-3.86) |
| **Intervention Scenario 2 (Progressive Universal):** Increase school entry assessment score of children with low Socioeconomic Index by 0.8 SD and others by 0.2 SD | | | | | |
| Overall | 40.0 (39.4-40.5) | 3.8% |  |  |  |
| *Socioeconomic Index* | |  |  |  |  |
| High | 19.7 (19.0-20.4) |  | ref |  | ref |
| Medium | 41.9 (41.3-42.5) |  | 22.2 | 0.0% | 2.13 (1.86-2.43) |
| Low | 58.5 (57.6-59.4) |  | 38.8 | 17.4% | 2.97 (2.59-3.40) |
| **Intervention Scenario 3 (Perverse Universal):** Increase school entry assessment score of children with high Socioeconomic Index by 0.8 SD and others by 0.2 SD | | | | | |
| Overall | 40.6 (40.0-41.3) | 2.4% |  |  |  |
| *Socioeconomic Index* | |  |  |  |  |
| High | 15.6 (15.1-16.1) |  | ref |  | ref |
| Medium | 41.9 (41.3-42.5) |  | 26.3 | -18.5% | 2.69 (2.31-3.12) |
| Low | 66.7 (65.7-67.6) |  | 51.1 | -8.7% | 4.28 (3.68-4.96) |
| **Intervention Scenario 4 (Pro-Equity Targeted):** Increase school entry assessment score by 0.8 SD only among children with low Socioeconomic Index | | | | | |
| Overall | 40.0 (39.4-40.5) | 3.8% |  |  |  |
| *Socioeconomic Index* | |  |  |  |  |
| High | 19.7 (19.0-20.4) |  | ref |  | ref |
| Medium | 41.9 (41.3-42.5) |  | 22.2 | 0.0% | 2.13 (1.86-2.43) |
| Low | 58.5 (57.6-59.4) |  | 38.8 | 17.4% | 2.97 (2.59-3.40) |

**Appendix S7**

**Sample sizes included for analyses**

ALSPAC children alive at 1 year
*n* = 13978

Multiply impute missing data

Missing data on covariates
*n* = 7474

Linked education data unavailable
*n* = 2214

Educational achievement data, 15-16 years
*n* = 11764

Complete cases
*n* = 4290

Imputed sample
*n* = 11764

Figure S7.1

*Sample sizes included for analyses*

**References for Appendices**

Barnett, W. S. (1995). Long-Term Effects of Early Childhood Programs on Cognitive and School Outcomes. *The Future of Children, 5,* 25-50.

Baron, R. M., & Kenny, D. A. (1986). The moderator-mediator variable distinction in social psychological research: conceptual, strategic, and statistical considerations. *Journal of Personality and Social Psychology, 51,* 1173-1182.

Campbell, F. A., Conti, G., Heckman, J. J., Moon, S. H., Pinto, R., Pungello, E., et al. (2014). Early Childhood Investments Substantially Boost Adult Health. *Science, 343,* 1478-1485.

Campbell, F. A., Pungello, E. P., Miller-Johnson, S., Burchinal, M., & Ramey, C. T. (2001). The development of cognitive and academic abilities: Growth curves from an early childhood educational experiment. *Developmental Psychology, 37,* 231-242.

Campbell, F. A., Ramey, C. T., Pungello, E., Sparling, J., & Miller-Johnson, S. (2002). Early childhood education: Young adult outcomes from the Abecedarian project. *Applied Developmental Science, 6,* 42-57.

Coffman, D. L., & Zhong, W. (2012). Assessing mediation using marginal structural models in the presence of confounding and moderation. *Psychological Methods, 17,* 642-664.

Cole, S. R., Platt, R. W., Schisterman, E. F., Chu, H., Westreich, D., Richardson, D., et al. (2010). Illustrating bias due to conditioning on a collider. *International Journal of Epidemiology, 39,* 417-420.

Currie, J. (2001). Early childhood education programs. *Journal of Economic Perspectives, 15,* 213-238.

Currie, J., & Thomas, D. (1995). Does Head Start make a difference? *American Economic Review, 85,* 341-364.

Currie, J., & Thomas, D. (2000). School quality and the longer-term effects of Head Start. *The Journal of Human Resources, 35,* 755-774.

D'Onise, K., Lynch, J. W., Sawyer, M. G., & McDermott, R. A. (2010). Can preschool improve child health outcomes? A systematic review. *Social Science & Medicine, 70,* 1423-1440.

D'Onise, K., McDermott, R. A., & Lynch, J. W. (2010). Does attendance at preschool affect adult health? A systematic review. *Public Health, 124,* 500-511.

Daniel, R., De Stavola, B., & Cousens, S. (2011). gformula: Estimating causal effects in the presence of time-varying confounding or mediation using the g-computation formula. *The Stata journal, 11,* 479-517.

Deming, D. (2009). Early childhood intervention and life-cycle skill development: Evidence from Head Start. *American Economic Journal: Applied Economics, 1,* 111-134.

Garces, E., Thomas, D., & Currie, J. (2002). Longer-term effects of Head Start. *The American Economic Review, 92,* 999-1012.

Gormley, W. T., Jr., Gayer, T., Phillips, D., & Dawson, B. (2005). The effects of universal Pre-K on cognitive development. *Developmental Psychology, 41,* 872-884.

Karoly, L. A., Greenwood, P. W., Everingham, S. S., Hoube, J., Kilburn, R., Rydell, C. P., et al. (1998). *Investing in our children. What We Know and Don't Know About the Costs and Benefits of Early Childhood Interventions*. Santa Monica: The RAND Corporation.

Karoly, L. A., Kilburn, M. R., & Cannon, J. S. (2005). *Early childhood interventions: proven results, future promise*. Santa Monica, CA: RAND Labor and Population.

Magnuson, K., Ruhm, C., & Waldfogel, J. (2007). Does prekindergarten improve school preparation and performance? *Economics of Education Review, 26,* 33-51.

NICHD Early Child Care Research Network. (2002). Early child care and children’s development prior to school entry: results from the NICHD Study of Early Child Care. *American Educational Research Journal, 39,* 133-164.

Olds, D. L., Kitzman, H., Cole, R., Robinson, J., Sidora, K., Luckey, D. W., et al. (2004). Effects of nurse home-visiting on maternal life course and child development: age 6 follow-up results of a randomized trial. *Pediatrics, 114,* 1550-1559.

Pearl, J. (2001). *Direct and indirect effects*. San Francisco, CA: Morgan Kaufmann.

Raver, C. C., Jones, S. M., Li-Grining, C., Zhai, F., Bub, K., & Pressler, E. (2011). CSRP's impact on low-income preschoolers' preacademic skills: self-regulation as a mediating mechanism. *Child Development, 82,* 362-378.

Robins, J. M., & Greenland, S. (1992). Identifiability and exchangeability for direct and indirect effects. *Epidemiology, 3,* 143-155.

Robins, J. M., Hernan, M. A., & Brumback, B. (2000). Marginal structural models and causal inference in epidemiology. *Epidemiology, 11,* 550-560.

Royston, P. (2004). Multiple imputation of missing values. *The Stata journal, 4,* 227-241.

Sammons, P., Elliot, K., Sylva, K., Melhuish, E., Siraj-Blatchford, I., & Taggart, B. (2004). The impact of pre-school on young children's cognitive attainments at entry to reception. *British Educational Research Journal, 30,* 691-712.

Schweinhart, L. J., Montie, J., Xiang, Z., Barnett, W. S., Belfield, C. R., & Nores, M. (2005). *Lifetime Effects: The High/Scope Perry Preschool Study Through Age 40*. Ypsilanti, MI: High/Scope Press.

VanderWeele, T. J. (2009). Marginal structural models for the estimation of direct and indirect effects. *Epidemiology, 20,* 18-26.

VanderWeele, T. J. (2010). Bias formulas for sensitivity analysis for direct and indirect effects. *Epidemiology, 21,* 540-551.

VanderWeele, T. J. (2012). Invited commentary: Structural equation models and epidemiologic analysis. *American Journal of Epidemiology, 176,* 608-612.

VanderWeele, T. J., & Vansteelandt, S. (2009). Conceptual issues concerning mediation, interventions and composition. *Statistics and Its Interface, 2,* 457-468.

Von Hippel, P. T. (2007). Regression with missing Y's: an improved strategy for analyzing multiply imputed data. *Sociological Methodology, 37,* 83-117.

Wong, V. C., Cook, T. D., Barnett, W. S., & Jung, K. (2008). An effectiveness-based evaluation of five state pre-kindergarten programs. *Journal of Policy Analysis and Management, 27,* 122-154.
